# New Appliances and Things Medical

**Published:** 1893-06-17

**Authors:** 


### NEW APPLIANCES AND THINGS MEDICAL.

[All preparations, appliances, novelties, etc., of which a notice is desired, should be sent for the Editor, to care of The Manager, 428, Strand, London, W.C.]

### "IZAL."

(Newton, Chambers, and Co., Thorncliffe, near Sheffield.)

The above is a new form of non-poisonous coal-tar disinfectant, discovered by Mr. J. H. Worrall, F.C.S., F.I.C., in the bye-products of the coke ovens at Thorncliffe. As sent out, it is a milky-looking fluid with a tar-like odour. It is stated to be insoluble in water, but forms an emulsion with it. It is also stated to be non-poisonous, and its dose for internal administration is given at from five to ten drops of the fluid in some suitable vehicle. It is also stated to be preferable to the different antiseptics in common use, inasmuch that it has (1) no caustic action on the skin; (2) is not poisonous; (3) being insoluble in water, it is not washed away from the part to which it may be applied; (4) it is stronger than carbolic acid, the disinfectant with which it is most easily comparable. According to the prospectus issued, a solution of 1-200 destroys in five minutes the germs of cholera, diphtheria, typhoid fever, pneumonia, anthrax, and glanders. We have experimented with it as an anti-putrescent and preservative, and find that with regard to these points it acts very efficiently. Internally in a case of flatulent dyspepsia, also, when given with a flavouring material, it certainly gave relief, but its taste is decidedly unpleasant. We think that "Izal" will be found a useful and reliable antiseptic fluid, and that it will be admirably adapted for general disinfectant purposes, such as washing and bathing fever patients, placing on the sheet over a fever room door, disinfecting typhoid and other poisonous stools, flushing drains and washing floors and sinks, and for the destruction of parasites of all kinds.

### DR. FARR'S CAMPHORETTED EUCALYPTUS MEDALLIONS.

(Foster and Co., Clements Lane, E.C.)

These medallions are manufactured for the purpose of carrying about on the person as a "safeguard" against infection. Whether they are likely to produce a state of non-infectious receptivity or antisepticize those who are carrying the materia morbi is improbable, but that they contain camphor and eucalyptus is certain, and the carrying of them may, like the "blessed word Mesopotamia," bring comfort to timid minds.

### VENTILATING SOCK.

(The Cushion Ventilating Sock Company, Sherwood Street, Nottingham).

To those who know the advantage of using socks within their boots as a means of added comfort and health in wet or cold weather, the socks before us will especially commend themselves. The ventilated cushion, which is arranged to fall exactly below the instep, is formed of a spring of thin pierced metal plates. These are not only hygienic, but are most comfortable, and afford such a support to the arch of the foot that they will be advantageously used on walking tours, and, above all, can be recommended to nurses. Some years ago boots with spring insteps were introduced, and although these were found to be very convenient, they were expensive, and never came into general use. The ventilating socks are excellent successors of the "Flexura" method, and as they are moderate in price and well made in leather or leather and felt, according to the warmth required, they most certainly merit a trial.

